# Delphinidin induces apoptosis via cleaved HDAC3-mediated p53 acetylation and oligomerization in prostate cancer cells

**DOI:** 10.18632/oncotarget.10790

**Published:** 2016-07-22

**Authors:** Mi-Hyeon Jeong, Hyeonseok Ko, Hyelin Jeon, Gi-Jun Sung, Soo-Yeon Park, Woo Jin Jun, Yoo-Hyun Lee, Jeongmin Lee, Sang-wook Lee, Ho-Geun Yoon, Kyung-Chul Choi

**Affiliations:** ^1^ Department of Biomedical Sciences, Asan Medical Center, University of Ulsan College of Medicine, Seoul, Korea; ^2^ Department of Pharmacology, University of Ulsan College of Medicine, Seoul, Korea; ^3^ Cell Dysfunction Research Center (CDRC), University of Ulsan College of Medicine, Seoul, South Korea; ^4^ Department of Biochemistry and Molecular Biology, Center for Chronic Metabolic Disease Research, Brain Korea 21 Plus Project for Medical Sciences, Severance Medical Research Institute, Yonsei University College of Medicine, Seoul, Korea; ^5^ Laboratory of Molecular Oncology, Cheil General Hospital & Women's Healthcare Center, Dankook University College of Medicine, Seoul, South Korea; ^6^ Department of Food and Nutrition, Chonnam National University, Gwangju, South Korea; ^7^ Department of Food Science and Nutrition, The University of Suwon, Kyunggi-do, South Korea; ^8^ Department of Medical Nutrition, Kyung Hee University, Yongin-si, Kyunggi-do, South Korea; ^9^ Department of Radiation Oncology, Asan Medical Center, University of Ulsan College of Medicine, Seoul, Korea

**Keywords:** delphinidin, p53, acetylation, HDAC3, apoptosis

## Abstract

Delphinidin is a major anthocyanidin compound found in various fruits. It has anti-inflammatory, anti-oxidant, and various other biological activities. In this study, we identified the epigenetic modulators that mediate the apoptotic effect of delphinidin in human prostate cancer cells. We found that treatment of LNCaP cells (a p53 wild-type, human prostate cancer cell line) with delphinidin increased caspase-3, −7, and −8 activity, whereas it decreased histone deacetylase activity. Among class I HDACs, the activity of HDAC3 was specifically inhibited by delphinidin. Moreover, the induction of apoptosis by delphinidin was dependent on caspase-mediated cleavage of HDAC3, which results in the acetylation and stabilization of p53. We also observed that delphinidin potently upregulated pro-apoptotic genes that are positively regulated by p53, and downregulated various anti-apoptotic genes. Taken together, these results show that delphinidin induces p53-mediated apoptosis by suppressing HDAC activity and activating p53 acetylation in human prostate cancer LNCaP cells. Therefore, delphinidin may be useful in the prevention of prostate cancer.

## INTRODUCTION

Prostate cancer is one the most widespread types of cancer. Until recently, it was the most common cancer among men in Western countries, whereas its incidence is increasing in Asian populations. In the United States alone, 186,320 new cases of prostate cancer will be diagnosed and a total of 28,660 deaths are predicted [1]. Prostate cancer is a slowly growing cancer and is usually detected at a late stage [2]. Most patients exhibit increased levels of specific cancer markers, such as the prostate specific antigen (PSA) which is secreted into blood circulation [3]. Because prostate cancer is initially androgen-dependent, androgen deprivation is commonly used to cause it to regress [4]. However, prostate cancers that are not cured by surgery eventually become androgen independent, thus resistant to anti-androgen therapy, and progress to highly aggressive metastatic cancer often leading to a patient's death [5]. Moreover, standard chemotherapy is rather ineffective for prostate cancer and has serious side effects due to its toxicity.

**Figure 1:**
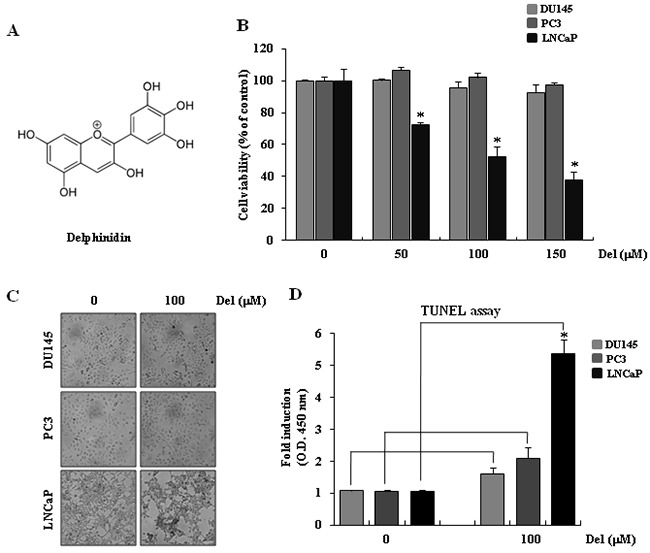
Delphinidin induces apoptosis in human prostate cancer LNCaP cells **A.** The structure of delphinidin. **B.** The cytotoxic effect of delphinidin on human prostate cancer cells, as measured using the MTT assay. Statistical significance was determined using Student's *t*-test; *P < 0.01 *versus* LNCaP cells not treated with delphinidin. **C.** Morphological changes of prostate cancer cells with or without delphinidin treatment. Cells were cultured in complete medium for 12 h. **D.** Dead cells were stained using TUNEL assay kits. As the dye is very positively charged, it cannot penetrate non-compromised cell membranes, thus it cannon enter and stain living cells. The arrow indicates dead cells. The data are expressed as mean ± SD (standard deviation) for triplicate measurements.

An alternative approach is the use of natural dietary compounds for either chemoprevention or chemotherapy. Two examples are resveratrol and EGCG, found in red wine and green tea, respectively. These have been demonstrated to be beneficial agents as they interfere with key processes involved in cancer development and progression. Among polyphenols, a promising dietary component is delphinidin (Figure 1A), one of the main anthocyanidins. Delphinidin is a diphenylpropane-based, polyphenolic ring structure-harboring compound that is naturally found in pomegranates, berries, grapes, beets, and eggplants [6]. Delphinidin possesses anti-cancer, anti-inflammatory, and anti-angiogenic properties. Recently published reports showed that it is able to inhibit the invasion of breast cancer cells [7]. Due to the factors mentioned above (emergence of androgen-independent prostate cancer cells, low efficiency and serious side effects of classic chemotherapy against this type of cancer), dietary chemoprevention and dietary therapy of prostate cancer are increasingly considered as a promising means to reduce the occurrence of this type of cancer, or reduce the side effects during its treatment, respectively [8, 9].

Histone deacetylases (HDACs) are widely expressed, highly conserved proteins. Eighteen human HDACs have been identified, which are grouped into four classes based on their homology to their respective yeast orthologs. Class I HDACs (1, 2, 3, and 8) are homologous to the yeast transcriptional regulator RPD3, class II HDACs (HDAC 4–7, 9, 10) are similar to Hda1, and class III HDACs (SIRTs 1–7) are NAD^+^-dependent histone deacetylases homologous Sir2 [10]. HDAC11 is quite different from the members of the other classes and has been placed in a fourth class. In addition to histone proteins, HDACs have many non-histone protein substrates, including p53, NF-kB, and STAT, which are important transcription factors regulating the expression of a large number of genes [11]. HDACs are involved in DNA replication, cell cycle progression, gene repression, cell proliferation, and tumorigenesis in various cells [12]. However, the roles of the various HDACs in cell proliferation and cell death are not yet fully established.

HDACs are important therapeutic targets in various human cancers, because they regulate the expression of p53 and its activation [13–16]. The p53 protein is a key transcription factor of tumor cell death signaling pathways as it regulates the expression of genes involved in apoptosis and cell cycle arrest [17, 18]. Another protein, MDM2, binds and ubiquinates p53, resulting in the rapid degradation of the latter. However, acetylation of p53 by two histone acetyltransferases (HATs), p300 and CBP, abrogates the ability of mdm2 to bind and ubiquinate p53, leading to p53 stabilization [19, 20]. As expected, deacetylation of p53 by HDACs has the opposite effect, i.e., it promotes its degradation. Among HDACs, HDAC3 localizes to the nucleus, cytoplasm, and plasma membrane. It is functionally distinct from other members of Class I HDACs [21] and exerts an important regulatory effect on the expression and function of p53. According to recent research, the cleavage of HDAC3 that takes place during apoptosis induced by chemotherapeutic agents, leads to the expression of p53-regulated pro-apoptotic genes [22].

In this study, we demonstrate that delphinidin induces apoptosis in prostate LNCaP cancer cells by inducing caspase-mediated HDAC3 cleavage that results in the acetylation and stabilization of p53. The activation of effector caspases during delphinidin-induced apoptosis is involved in the cleavage and inactivation of HDAC3, whereas the downregulation of HDAC3 activity leads to the oligomerization of p53 in human prostate cancer LNCaP cells. Moreover, delphinidin-induced apoptosis is accompanied by the upregulation of pro-apoptotic genes such as *Bax* and *p21*. To sum up, we demonstrated that treatment of LNCaP cells with delphinidin significantly enhanced p53 stabilization and p53 acetylation. Delphinidin appears to serve as a multifunctional anti-tumor and HDAC-inhibition agent in various types of human cancer.

## RESULTS

### Delphinidin induces apoptosis in human LNCaP prostate cancer cells

Delphinidin, the major anthocyanidin compound in pigmented fruits and vegetables, possesses a diphenylpropane-based, polyphenolic-ring structure and has anti-inflammatory, anti-oxidant, and anti-angiogenic activity (Figure 1A). Previously published reports also suggest that treatment of prostate cancer cells with delphinidin results in an efficient, dose-dependent inhibition of cell growth. Hafeez et al. (2008) of the University of Wisconsin observed that whereas delphinidin exerts a differential dose response effect on the growth of prostate cancer cells, it did not affect the viability of normal prostate epithelial cells [6].

To examine the mechanism of delphinidin-induced apoptosis, we first examined the cytotoxic effect of delphinidin on human prostate cancer cells using a cell viability assay. Delphinidin did not affect the viability of Du145 or PC3 cells at any of the used concentrations, whereas it had a clear dose-dependent cytotoxic effect on LNCaP cells. (Figure 1B).

In order to confirm that the aforementioned changes in viability were a result of delphinidin-induced apoptosis, we microscopically observed and applied the TUNEL assay on prostate cancer cell lines after a 12-hour treatment with 100-μM delphinidin. As shown in Figure 1C, delphinidin-treated LNCaP cells displayed changes in morphology that relate to apoptosis, whereas Du145 and PC3 cells did not exhibit similar changes in cellular morphology. The TUNEL assay can be used to detect dead or dying cells exhibiting DNA fragmentation, which is a sign of apoptosis. After delphinidin-treated cells were stained, the dead cells were measured in a multiplex microplate reader (Figure 1D). As seen in Figure 1D, treatment of LNCaP cells with delphinidin resulted in the appearance of labeled dead cells. In contrast, no stained cells were detected in delphinidin-treated Du145 and PC3 cultures. Therefore, both the morphological observations and the TUNEL assay results indicate that delphinidin induces apoptosis of LNCaP cells. The fact that LNCaP cells are wt-p53 positive, while the two unaffected cancer cell lines, Du145 or PC3, do not express functional p53, suggests that delphinidin induces apoptosis in a p53-dependent fashion.

### Delphinidin induces caspase-dependent apoptosis in LNCaP cells

**Figure 2:**
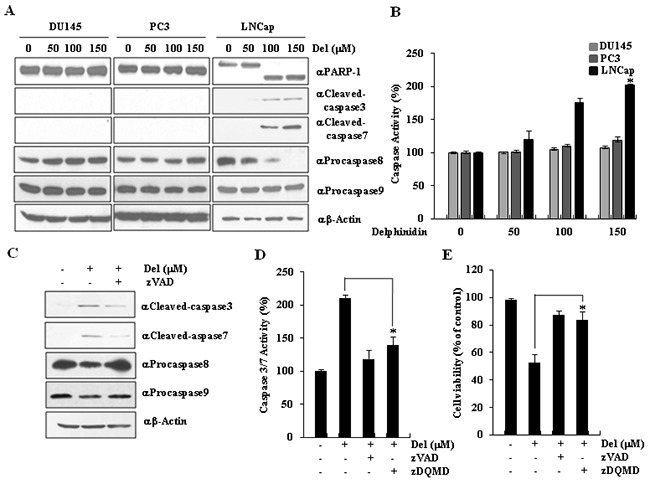
Delphinidin induces caspase-dependent apoptosis in LNCaP cells **A.** Delphinidin induced the activation of effector caspases. LNCaP cells were treated with delphinidin (100 μM) for 12 h. The expression of caspase-8, caspase-9, and the levels of cleaved caspase-3, cleaved caspase-7, and cleaved PARP-1 were activated by delphinidin treatment, as determined by western blot analysis. **B.** Delphinidin increased caspase activity in LNCaP cells. The total caspase activity in prostate cancer cells treated with delphinidin was measured. **C.** Delphinidin-induced apoptosis was suppressed by caspase inhibitor zVAD. LNCaP cells were pretreated for 30 min with or without zVAD (40 μM); afterwards, they were treated with delphinidin (100 μM) for 12 h. **D.** Delphinidin significantly induced the activation of caspases in LNCaP cells. LNCaP cells were pretreated for 30 min with zVAD (40 μM) or zDQMD; afterwards, they were treated with delphinidin (100 μM) for 12 h. After incubation, the activity of caspase-3/7 was inhibited by zVAD or zDQMD treatment. **E.** The cytotoxicity effect of delphinidin was reduced by caspase inhibitors. Cell viability was measured using the MTT assay. All data are expressed as the mean ± SD for triplicate measurements. Statistical significance was determined using Student's *t*-test; *P < 0.01 *versus* LNCaP cells that were not treated with delphinidin.

Caspases play a major role in cancer cell apoptosis. Caspases-8 and-9 directly or indirectly activate the effector caspases-3 and −7, which serve as key factors in the apoptosis signaling pathway [23]. To investigate the mechanism of delphinidin-induced apoptosis in LNCaP cells, we examined the activation of caspases-3, −7, −8, and −9, as well as the cleavage of PARP-1 in the cytosol. PARP-1 is a substrate of effector caspases, so the presence or absence of the cleaved form is a clear indication of whether or not caspases play an essential role in delphinidin-induced apoptosis of LNCaP cells. Prostate cancer cells were incubated with various concentrations of delphinidin (50, 100, and 150 mM) for 24 h, while non-treated cells served as controls. In LNCaP cells, delphinidin treatment led to an increase in the expression of caspases-8 and to higher levels of the cleaved forms of caspase-3, caspase-7, and PARP-1, whereas no changes were observed in Du145 and PC3 cells (Figure 2A). These results clearly suggest that the delphinidin-induced apoptosis of LNCaP cells was initiated by caspase-8 activation. Caspase-8 cleaved caspases-3 and −7 activating them. Truly, our experiments showed that delphinidin-induced apoptosis of LNCaP cells was accompanied by a significant increase in caspase activity (Figure 2B). In turn, the activated effector caspases cleaved their substrate, PARP-1. As a whole, our results suggest that delphinidin-induced apoptosis in LNCap is mediated by caspase activation.

To confirm the role of the caspase cascade in the delphinidin-induced apoptosis of LNCaP cells, we tried to inhibit apoptosis by blocking caspase activation with a general caspase inhibitor (zVAD). LNCaP cells were incubated with 100 μM delphinidin for 24 h, in the presence or absence of zVAD. As shown in Figure 2C, caspase activation in delphinidin-treated LNCaP cells was inhibited by zVAD treatment. We proceeded to examine the effect of zVAD, as well as the effect of a specific inhibitor of caspases-3 and −7, zDQMD, on the delphinidin-induced apoptosis of LNCaP cells. The caspase-3/-7 activity analysis showed that the delphinidin-induced activation of these two caspases was significantly inhibited by zDQMD (Figure 2D). To evaluate whether the inhibition of caspases-3 and −7 decreases the cytotoxicity that is caused by delphinidin treatment, we performed another viability assay. The inhibitor effectively blocked caspase activity and reduced cytotoxicity. Therefore, the inactivation of caspases by zVAD or zDQMD dramatically inhibits delphinidin-induced apoptosis in LNCaP cells (Figure 2E). Taken together, these results strongly suggest that delphinidin promotes apoptosis in these cells by activating the effector caspases-3 and −7.

### Delphinidin antagonizes HDAC3 activity and increases the cytotoxicity of LNCaP cells

Histone modifications regulate the activation and stabilization of histones and non-histone proteins. HATs and HDACs dynamically regulate chromatin remodeling, histone modification, and gene expression. The main function of HDACs is the repression of gene transcription via deacetylation of lysine residues in histones or non-histones [24]. HDAC3 is known to regulate the transcription of genes through its effect on chromatin conformation [25, 26], and its functions have been demonstrated in many research studies. Recently, we reported the existence of three novel HDAC3-interacting partners, L3MBTL1, CREB3, and PDCD5, and described roles of HDAC3 in epigenetic regulation mediated by these proteins [27]. It is our belief that HDAC3 is an important regulatory factor of proliferation, growth homeostasis, and apoptosis of cancer cells during various cellular events.

**Figure 3:**
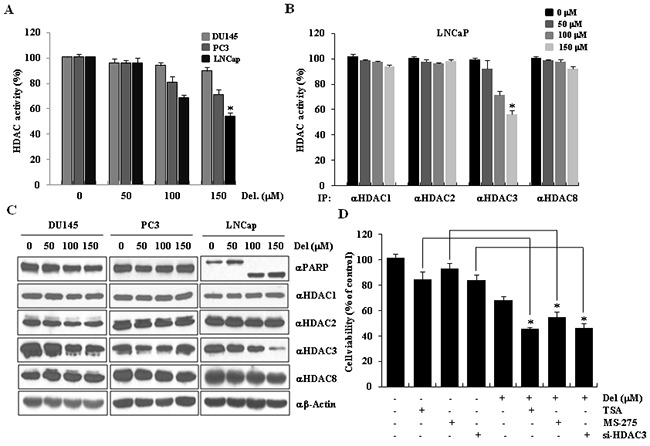
HDAC3 activity was downregulated during delphinidin-induced apoptosis in LNCaP cells **A.** Delphinidin reduces total HDAC activity. Prostate cancer cells were treated with various concentrations of delphinidin (50, 100, and 150 μM) for 24 h. Non-treated cells were used as controls. Statistical significance was determined using Student's *t*-test; *P < 0.01 *versus* controls. **B.** Delphinidin inhibits HDAC3 activity. HDAC activity assays were performed using a colorimetric HDAC activity assay kit. The data are expressed as mean ± SD for triplicate measurements. Statistical significance was determined using Student's *t*-test; *P < 0.05 *versus* non-treated controls. **C.** HDAC3 expression was reduced by delphinidin treatment. LNCaP cells were treated with delphinidin. Cell lysates were processed for western blot analysis using the indicated antibodies. **D.** The viability of LNCaP cells was decreased by HDAC inhibitors and HDAC3 siRNA. LNCaP cells were treated with delphinidin and TSA (MS-275 or HDAC3 siRNA) for 12 h. The cell viability was measured using the MTT assay. All data are expressed as the mean ± SD for triplicates. Statistical significance was determined using Student's *t*-test; *P < 0.01 *versus* LNCaP cells that were not treated with delphinidin.

To determine whether the mechanism through which delphinidin induces apoptosis in LNCaP cells includes downregulation of HDAC3 activity; we measured total HDAC activity in cells treated with delphinidin. HDAC activity was decreased by delphinidin in both PC3 cells and LNCaP cells. However, the reduction was much greater in LNCaP cells. (Figure 3A). We then examined whether delphinidin is a global or a specific HDAC inhibitor. As seen in Figure 3B, delphinidin reduced the activity of HDAC3 activity to a greater extent compared to the activity of the other class I HDACs. We concluded that delphinidin induced apoptosis of LNCaP cells via the specific inhibition of HDAC3 activity. To examine the mechanism through which the reduction of HDAC3 activity takes place, we determined the protein levels of class I HDACs and PARP-1 in delphinidin-treated cancer cells using western blot analysis. As shown in Figure 3C, delphinidin treatment dramatically reduced HDAC3 expression and induced PARP-1 cleavage in a dose-dependent manner. These data suggest that delphinidin induces apoptosis in LNCaP cells through the suppression of HDAC3 expression and deacetylase activity. To confirm that the suppression of HDAC activity leads to apoptosis, we examined the viability of LNCaP cells that were incubated with 100 μM delphinidin alone, or together with either TSA, a general HDAC inhibitor, or MS-275, a specific HDAC1/3 inhibitor. As expected, in both cases of co-treatment the viability of LNCaP cells was lower compared to the viability of cells treated with delphinidin alone. We also examined whether knocking down HDAC3 using siRNA enhanced the apoptotic effect of delphinidin in LNCaP cells. As shown in Figure 3D, HDAC3-knocked down cells treated with delphinidin displayed a significantly lower viability compared to normally expressing HDAC3 cells treated with delphinidin alone or co-treated with delphinidin and HDAC inhibitors. Thus, HDAC3 RNAi-mediated knockdown can enhance the apoptotic effect of delphinidin on LNCaP cells. Taken together, our findings reveal that HDAC3 is a critical factor in delphinidin-induced apoptosis in human prostate cancer LNCaP cells.

### Delphinidin-induced HDAC3 cleavage leads to p53 acetylation in LNCaP cells

**Figure 4:**
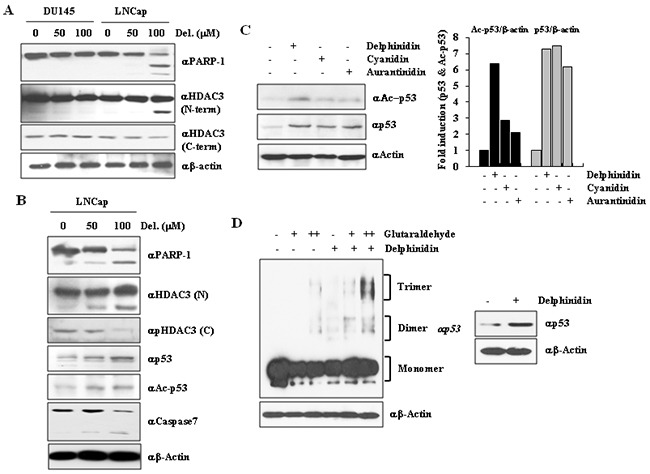
HDAC3 cleavage induces p53 acetylation and oligomerization **A.** HDAC3 was cleaved because of delphinidin treatment. LNCaP cells were treated with delphinidin (100 μM). HDAC3 cleavage was then detected by western blot analysis using the indicated HDAC3 antibody. **B.** Delphinidin induced p53 acetylation through HDAC3 inactivation. Delphinidin–treated LNCaP cell lysates were analyzed by western blot analysis using the indicated antibodies. **C.** Delphinidin induced hyperacetylation of p53, and anthocyanidins upregulated p53 expression. **D.** Delphinidin induced p53 multimerization. Delphinidin-treated LNCaP cells were extracted using lysis buffer, and cell extracts were incubated with glutaraldehyde for 20 min at 37 °C. Samples underwent western blot analysis using an anti-p53 antibody.

Various studies have reported that LNCaP cells harbor two functional alleles encoding wild-type p53, whereas DU145 cells express a modified p53 protein with two point mutations (Phe223Leu and Val274Phe) and PC3 cells are deficient in functional p53 protein production. To further support the finding that delphinidin-induced apoptosis is mediated by a downregulation of HDAC3 expression and activity, we examined the effects of different concentrations of delphinidin on p53 acetylation. According to previous reports [28, 29], HDAC3 is cleaved because of sorbitol-induced caspase-7 activation, or during FasL-mediated apoptosis. As p53 is one of the targets of the deacetylases activity of HDAC3, the cleavage of HDAC3 during apoptosis results in an increase in p53 acetylation, whereas the transcription of pro-apoptotic genes is activated. Based on these reports, we proceeded to examine whether an increase in p53 acetylation due to HDAC3 cleavage is also taking place in delphinidin-induced apoptosis. LNCaP cells and DU145 cells treated with delphinidin (50 and 100 mM), as well as untreated controls, were examined using western blot analysis. The apoptotic effect of delphinidin in LNCaP cells was accompanied by cleavage of both PARP-1 and HDAC3 (Figure 4A). The cleaved form of HDAC3 can be detected using an anti-HDAC3 antibody raised against the N-terminal region of anti-HDAC3, whereas antibodies recognizing the C-terminal region are not able to bind to cleaved HDAC3.

We then determined whether p53 acetylation and stability were enhanced by HDAC3 cleavage during delphinidin-induced apoptosis. LNCaP cells were treated with different doses of delphinidin for 24 h. As shown in Figure 4B, delphinidin increased p53 acetylation and stability, whereas HDAC3 was cleaved by activated caspase-7 in delphinidin-treated LNCaP cells, in which apoptosis was induced. We also examined the effect of two other anthocyanidins, cyanidin and aurantinidin, on the p53 acetylation status. As shown in Figure 4C, delphinidin increased p53 acetylation and stability to a greater extent compared to that by cyanidin and aurantinidin.

To demonstrate that endogenous p53 is stabilized for activation in response to delphinidin, p53 was immunoprecipitated from lysates of LNCaP cells treated with delphinidin and/or glutaraldehyde (a protein-protein crossinker). Proteins were immunoprecipitated for 20 min at 37°C and detected by western blot analysis using an anti-p53 antibody. These results show that the stabilization and activation of p53 were induced by enhancing the oligomerization of p53 (Figure 4D), whereas it is known that oligomerization of p53 is essential for enhancing p53 activity for DNA binding, protein-protein interactions, post-translational modification, and stabilization [30]. As a whole, these findings suggest that delphinidin induced p53-mediated apoptosis in LNCaP cells through p53 oligomerization.

### Delphinidin-induced apoptosis of LNCaP cells is mediated by activation of p53 and inactivation of HDAC3

**Figure 5:**
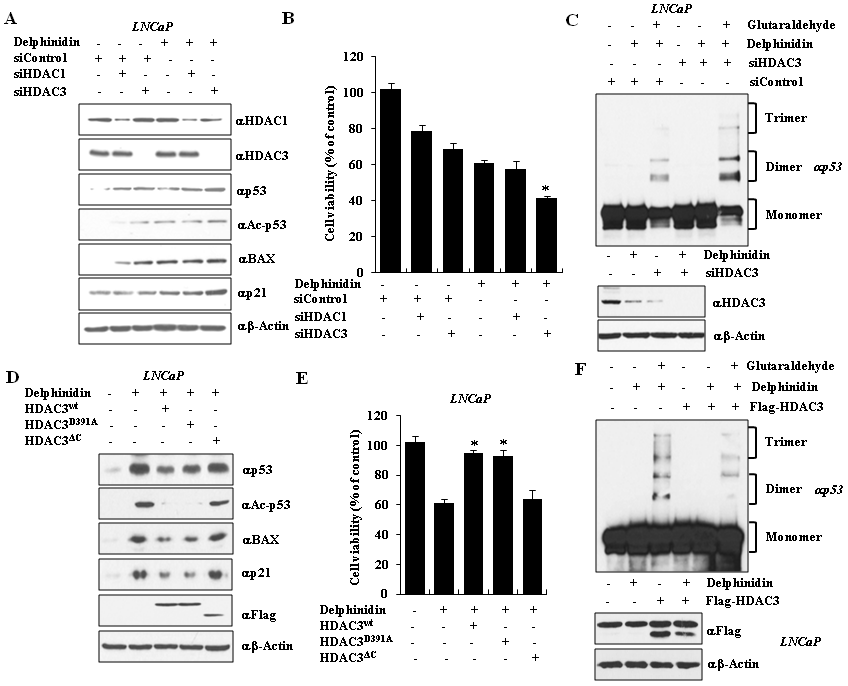
The silencing or C-terminal mutation of HDAC3 activates the expression of p21 and BAX by inducing oligomerization of p53 **A.** HDAC3 silencing increased expression of BAX and p21 in delphinidin–treated LNCaP cells. HDAC3 siRNA was transfected into LNCaP cells with delphinidin. **B.** Co-treatment with delphinidin and HDAC3 siRNA reduced cell viability, which was measured using the MTT assay. All data are expressed as the mean ± SD for triplicate measurements. Statistical significance was determined using Student's *t*-test; *P < 0.01 *versus* HDAC3 siRNA-treated LNCaP cells. **C.** HDAC3 silencing enhanced p53 oligomerization. **D, E.** HDAC3^wt^ and HDAC3^D391A^ inhibited cell viability during delphinidin-mediated apoptosis. **D.** Delphinidin–treated LNCaP cell extracts were analyzed by western blot analysis using the indicated antibodies. **E.** The cell viability was measured using the MTT assay. All data are expressed as the mean ± SD for triplicates. Statistical significance was determined using Student's *t*-test; *P < 0.01 *versus* delphinidin-treated LNCaP cells. **F.** HDAC3 over-expression inhibited p53 oligomerization.

According to recent reports, p53-dependent apoptosis of cancer cells is regulated by post-translational modification of p53, including acetylation. Therefore, we examined the results of knocking down HDAC1 or HDAC3 on the stability of p53, on the p53 acetylation levels, as well as on the expression of the pro-apoptotic proteins p21 and BAX (which is regulated by p53), in delphinidin-treated LNCaP cells. Treatment of LNCaP cells with delphinidin and si-HDAC1 or si-HDAC3 increased total p53 and acetylated-p53 protein levels, as well as the expression of p21 and BAX proteins, whereas the increase was greater when HDAC3 was knocked down (Figure 5A). We also performed a cell viability assay, where treatment of LNCaP cells with si-HDAC3 and delphinidin led to increased cytotoxicity. We conclude that HDAC3 inhibition enhances the apoptotic effect of delphinidin in LNCaP cells.

To demonstrate that endogenous p53 undergoes oligomerization in delphinidin-treated cells, p53 was immunoprecipitated from cell lysates of LNCaP cells treated with delphinidin and/or si-HDAC3, and visualized using western blot analysis. As seen in Figure 5C, p53 oligomerization was increased in delphinidin-treated LNCaP HDAC3 knockdown cells, compared to the delphinidin-treated cells whose HDAC3 expression had not been reduced by si-RNA treatment. We conclude that HDAC3 is directly involved in p53-dependent apoptosis. To further study the effect of HDAC3 on p53 stability in the presence of delphinidin, we overexpressed Flag-fused proteins containing wild-type HDAC3, or a point-mutation of the protein (HDAC3^D309A^), or a C-terminal deletion mutant (HDAC3^ΔC^). Overexpression of wild-type HDAC3 and HDAC3^D309A^ inhibited the ability of delphinidin to induce acetylation and stabilization of p53. The expression of p21 and BAX proteins was reduced (Figure 5D) and the cells became resistant to delphinidin-induced cytotoxicity (Figure 5E). In contrast, overexpression of HDAC3^ΔC^ did not inhibit the effects of delphinidin on p53 levels, p53 acetylation, p21 and BAX expression, or cytotoxicity (Figure 5D, Figure 5E). We next examined whether the p53 oligomerization caused by delphinidin is negatively regulated by HDAC3. As shown in Figure 5F, HDAC3 overexpression is able to inhibit the oligomerization of p53 in delphinine-treated LNCaP cells.

### Delphinidin-induced HDAC3 downregulation leads LNCaP cells to apoptosis via the activation of p53 target genes

**Figure 6:**
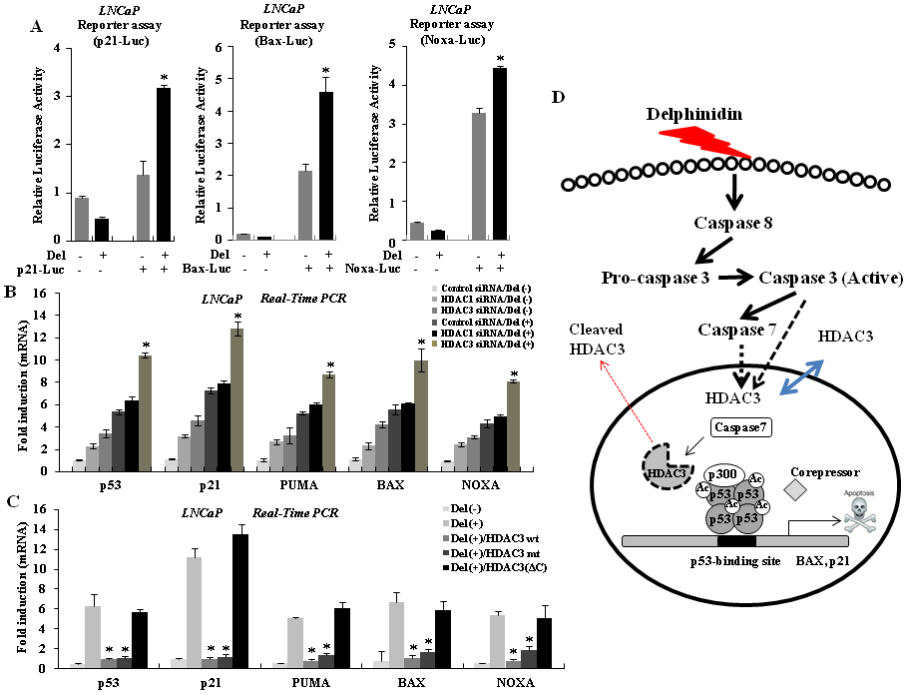
HDAC3 regulates the expression of p53 target genes **A.** Delphinidin increases the transcriptional activity of p53 target genes. LNCaP cells were transiently co-transfected with reporter constructs p21-Luc, Bax-Luc, or Noxa-Luc, and with pSV40 plasmids. After delphinidin treatment, cells were harvested and extracted, and dual luciferase activity was measured. Reporter activities were normalized relative to *Renilla* luciferase activities and were expressed as mean ± SD for triplicate measurements. Statistical significance was determined using Student's *t*-test; *P < 0.01 *versus* LNCaP cells that had not been treated with delphinidin. **B.** Knock-down of HDAC3 with delphinidin treatment enhances the expression of p53 target genes. Statistical significance was determined using Student's *t*-test; *P < 0.01 *versus* delphinidin-treated LNCaP cells. **C.** Over-expression of HDAC3 represses the expression of p53 target genes, but not expression of HDAC3^ΔC^. The expression level of each gene was analyzed by qRT-PCR using the total mRNA from LNCaP cells treated with delphinidin and with/without HDAC3. The data are expressed as the mean ± SD for triplicate measurements. Statistical significance was determined using Student's *t*-test; *P < 0.01 *versus* delphinidin-treated LNCaP cells. **D.** A model based on our findings. Treatment with delphinidin increases the expression of active caspases in LNCaP cells. The active caspases then induce HDAC3 cleavage, resulting in p53 acetylation, activation, and oligomerization. Importantly, delphinidin accelerates the hyperacetylation of p53 in order to activate p53-related apoptosis markers after HDAC3 inactivation by caspase activation.

Recent studies have demonstrated that the cleavage of HDAC3 in the apoptotic signaling pathway enhances the expression of p53-regulated pro-apoptotic genes [22, 29]. To examine the activation of the transcription of p53 target genes because of delphinidin treatment, we created reporter plasmids consisting of the promoters of three genes, *p21*, *Bax*, and *Noxa*, and the coding sequence of luciferase. The promoters of these three genes contain p53 binding elements and are activated by p53. We transiently transfected LNCaP cells with the reporter plasmids and treated the cells with delphinidin for 24 h. As shown in Figure 6A, delphinidin treatment led to the activation of all three promoters. We conclude that delphinidin-induced apoptosis in LNCaP cells is accompanied by the transcriptional activation of p53-upregulated pro-apoptotic genes. We next examined the role of HDAC3 in the expression of pro-apoptotic genes. HDAC3 knockdown dramatically enhanced the expression of *p53* and the pro-apoptotic genes *Bax*, *p21*, *Puma*, and *Noxa*. HDAC1 knockdown did not have a similar effect on the expression of these genes (Figure 6B). We also performed experiments where wild-type and mutant forms of HDAC3 were overexpressed. As shown in Figure 6C, overexpressing of HDAC3^wt^ and HDAC3^D391A^ suppressed the delphinidin-induced transcription of pro-apoptotic genes, whereas HDAC3^ΔC^ did not affect the transcription of these genes. Therefore, our results demonstrate that HDAC3 is cleaved by effector-caspases in response to delphinidin-initiated cell death signaling, resulting in the transcriptional activation of pro-apoptotic target genes, including *Puma*, *Bax*, and *Noxa*. As shown in Figure 6D, delphinidin treatment of LNCaP cells results in activation of effector caspases, whereas activated caspase-3 cleaves HDAC3. These results suggest that HDAC3 cleavage leads to the hyperacetylation and oligomerization of p53, as well as to the transcriptional activation of p53 target genes during delphinidin-induced apoptosis in p53 wild-type human prostate cancer cells.

## DISCUSSION

Prostate cancer is the second most common type of cancer in men worldwide and its incidence is continuously increasing. Recently, a worldwide pharmaceutical company started to develop novel plant-based natural compounds for the prevention and treatment of prostate cancer [6, 31].

Anthocyanidins are flavonoid polyphenols that are abundant in fruits and vegetables [32]. Several published studies have demonstrated the potential of flavonoids as anti-tumor chemopreventive and chemotherapeutic agents. Flavonoids have also been associated with beneficial effects in the treatment of diabetic retinopathy, atherosclerosis, and various microvascular diseases, which are attributed to their potential anti-inflammatory properties [32]. Delphinidin is an anthocyanidins with potent anti-oxidant, anti-inflammatory, and anti-cancer properties. It may aid in the development of mechanism-based cancer-prevention approaches [33].

In this study, we demonstrated the chemotherapeutic effects of delphinidin by studying its pro-apoptotic effect on human prostate cancer cells. Treatment of LNCaP cells with delphinidin reduced cell viability (Figure 1) by inducing apoptosis (Figure 2). Delphinidin treatment led to the activation of initiator caspases and effector caspases, and resulted in the cleavage of PARP-1 and HDAC3 in LNCaP cells (Figure 3), as well as in a significant increase in the expression of the pro-apoptotic proteins Bax, PUMA, and p21 (Figure 6). Our results also show that the stability of p53 determines the fate of cancer cells in patients being treated with delphinidin or with a combination of delphinidin and other agents.

HDACs are transcriptional co-repressors that regulate cell cycle arrest, cell differentiation, and apoptosis in cancer cell lines [34]. Inhibition of HDACs has been shown to enhance tumorigenesis in a variety of cancer cell types [35]. Clinical trials of HDAC inhibitors for the treatment of both hematopoietic and solid tumors are currently underway [36]. HDACs have a physiological role in the maintenance of cell proliferation, cell survival, and the suppression of intestinal and epithelial cell maturation. Therefore, treatment with HDAC inhibitors induces growth arrest, maturation, and apoptosis of cancer cell lines. Among HDACs, HDAC1 and HDAC2 have been demonstrated to play a role in repressing intestinal cell maturation and promoting cell proliferation and survival [37, 38]. In this study, we investigated the role of HDAC3 in cell survival and apoptosis; we observed increased apoptotic rates in HDAC3 knockdown LNCaP cells treated with delphinidin. We also demonstrated that HDAC3 cleavage in delphinidin-induced apoptosis results in the p53-dependent activation of the expression of *p21* and *Bax*, whereas overexpression of HDAC3 significantly reduced the delphinidin-induced activation of the *p21, Bax*, and *Noxa* promoters. Conversely, knockdown of HDAC3 enhanced the activating effect of delphinidin on these promoters. Moreover, HDAC3 cleavage reduced cell growth and cell survival, and mediated the dose-dependent apoptotic effect of delphinidin.

The p53 protein is very important for the regulation of viability and genomic stability of cancer cells [39], because it activates major pro-apoptotic genes; therefore, mutations of p53 have been reported in a variety of human cancers. The ability of p53 to exert its function is efficiently regulated by specific modifications, cellular localization, and cell-cycle phase [40]. Acetylation of lysine residues in the p53 C-terminus has been shown to block its interaction with MDM2, inhibiting its ubiquitination and degradation. Therefore, acetylation of p53 is a key regulatory factor for p53 stability and activity during cancer cell apoptosis. Acetylation of p53 takes place at specific lysine residues and is carried out by acetyltransferases such as p300 and CBP. The status of acetylated p53 is known to be regulated by various HDACs [41]. Indeed, deacetylation of p53 by HDAC3 is required for repressing the hyperacetylation of p53 during the apoptosis of cancer cells. In this study, we have demonstrated that the inactivation of HDAC3 increases the levels of p53 acetylation, its stability and causes its oligomerization, leading to apoptosis. Our data suggests that delphinidin treatment leads to caspase-mediated HDAC3 cleavage, resulting in the hyperacetylation of p53.

To sum up, we investigated the apoptotic effect of delphinidin on prostate cancer cells and demonstrated that the delphinidin-induced apoptosis is achieved through the activation of p53. Caspases have been shown to be involved in the induction of apoptosis by delphinidin. Indeed, our results show that the inactivation and cleavage of HDAC3 is a result of the activation of effector caspases. We also ascertained that delphinidin-induced apoptosis could be blocked by inhibiting HDAC3 cleavage with specific caspase-3/-7 inhibitors. Our results strongly suggest that delphinidin, as well as other HDAC inhibitors, should be considered as potential agents for the treatment of human prostate cancer.

## MATERIALS AND METHODS

### Cell culture and reagents

The human prostate cancer cell lines LNCaP, PC3, and DU145, were obtained from the American Type Culture Collection and were cultured in RPMI supplemented with 10% fetal bovine serum (FBS) and 1% antibiotics-antimycotics in a humidified 5% CO_2_ atmosphere at 37 °C. Cells were treated with delphinidin (Sigma-Aldrich, St. Louis, MD, USA). RPMI, antibiotics-antimycotics, FBS, and the Lipofectamine 2000 transfection reagent were purchased from Thermo-Fischer Scientific (Waltham, MA, USA).

### Cell viability assay

Cell viability was determined using the conventional MTT reduction assay. Briefly, 5 × 10^3^ – 1 × 10^4^ cells were seeded per well in 96-well plates. After a 12-hour incubation, cells were incubated with or without delphinidin (50, 100, or 150 mM) for another 24 h. Afterwards, cells were treated with 15 mL of MTT solution (2 mg/mL) for 90 min at 37 °C. The absorbance was measured at 570 nm in a Model 550 micro plate reader (BIO-RAD Laboratories, Hercules, CA, USA), whereas a reference wavelength of 630 nm was used. All results of MTT assays are presented as the means (± standard deviation) of three independent experiments.

### RNAi experiments

The siRNAs used in this study were purchased from Bioneer Corporation (Daejeon, Korea), and had the following sequences: HDAC1 siRNA, 5′-GAGUCAAAACAGAGGAUGA-3′; HDAC3 siRNA, 5′-GAGCUUCAAUAUCCCUCUA-3′. After a 12-hour incubation of LNCaP cells in RPMI not containing FBS or antibiotics, transfection was carried out using Lipofectamine 2000 with 100 pmol non-specific siRNA, HDAC1 siRNA, or HDAC3 siRNA, according to the manufacturer's instructions. After 4 h, the medium was changed and cells were incubated for another 2 days.

### TUNEL assay

TUNEL assay was performed using the HT Titer TACS Assay Kit (Trevigen, Gaithersburg, MD, USA) according to the manufacturer's instructions. LNCaP cells were fixed with 3.7% buffered formaldehyde solution for 5 min and washed with PBS. Cells were then permeabilized in 100% methanol for 20 min, washed twice with PBS, digested with proteinase K for 15 min, quenched with 3% hydrogen peroxide, washed with distilled water, labeled and incubated with deoxynucleotidyl transferase at 37 °C for 90 min, and then treated with stop buffer. Afterwards, cells were incubated in the presence of TACS-Sapphire substrate for 30 min. The colorimetric reaction was stopped with 0.2 N HCl and measured in a multiplex microplate reader at 450 nm absorbance.

### Caspase activity assay

Delphinidin-induced caspase activation was evaluated using Caspase-Glo −3/-7 and total caspase kits (Promega, Madison, WI, USA) according to the manufacturer's instructions. Briefly, LNCaP, PC3, and DU145 cells (4,000 cells per well in 100 μL media) were plated in 96-well, white-walled, clear-bottom plates (Lonza, Basel, Switzerland). The cells were then treated with delphinidin. After 24 h, 100 μL of assay reagent was added to each well. The plate was incubated in the dark for 60 min, and afterwards the luminescence was measured using a SpectraMAX 250 Optima plate reader (Molecular Devices, Sunnyvale, CA, USA).

### HDAC activity assays

The histone deacetylase (HDAC) activity assay was carried out using a commercially available kit (Biovision, San Franscisco, CA, USA) according to the manufacturer's instructions. In order to perform activity assays for specific HDACs, HDAC1, HDAC2, HDAC3, and HDAC8 proteins were first immunoprecipitated using anti-HDAC1, anti-HDAC2, anti-HDAC3, and anti-HDAC8 from LNCaP nuclear extracts. Immunoprecipitated complexes were collected and washed with HDAC assay buffer (50 mM Tris pH 8.0, 10% glycerol, 0.1 mM EDTA).

### Western blot analysis

After a 12-hour treatment with delphinidin, prostate cancer cells were washed with cold PBS, scraped off, and harvested. Cell extracts were prepared with lysis buffer (50 mM Tris-Cl pH 7.5, 150 mM sodium chloride, 1% NP40, 10 mM sodium fluoride, 10 mM sodium pyrophosphate, and protease inhibitors just before use) and incubated for 30 min on ice. The lysates were centrifuged at 20,000 *× g* for 10 min at 4 °C. Cell lysate proteins were separated by SDS-PAGE using 8% and 12% gels, and then transferred to nitrocellulose membranes. The membranes were blocked by incubating them for 2 h in 5% *w/v* non-fat milk in PBST. Blocked membranes were incubated with the primary antibody for 2 h or overnight at 4 °C. After washing with 1X PBST, the membranes were incubated with the secondary HRP-conjugated antibody for 1 h. The membranes were then subjected to western blot analysis and were visualized in the developer apparatus. The antibody against HDAC3 (N-terminal detection antibody) was manufactured by ATgen (Seongnam, Korea). HDAC1, HDAC2, HDA3, HDAC8, caspase-3, caspase-7, caspase-8, caspase-9, p53, p21, BAX, and PARP-1 antibodies were purchased from Santa Cruz Biotechnology (Dallas, TX, USA). Acetylated-p53 (Lys373) antibody was purchased from Merck Millipore (Darmstadt, Germany). Flag and β-actin antibodies were purchased from Sigma-Aldrich. Secondary anti-mouse and anti-rabbit antibodies were purchased from Pierce (Rockford, IL, USA).

### RNA extraction and quantitative reverse transcription-PCR

Total RNA was isolated with the RNA Easy-spin kit (Intron, Korea) according to the manufacturer's instructions, and reverse transcribed with random primers using the StrataScript reverse transcriptase kit (Stratagene, La Jolla, CA, USA) according to the manufacturer's protocol. All samples were normalized to *GAPDH* and expressed as fold changes. All reactions were done in triplicate. The relative expression levels and standard deviations were calculated using the comparative quantification method. The primers used were the following: *p53*, 5′- CCCAAGCAATGGATGATTTGA-3′ and 5′-GGCATTCTGGGAGCTTCATCT-3′; *p21*, 5′- GTGG AGAGCATTCCATCCCT-3′ and 5′- TGGATGCAGCT TCCTCTCTG-3′; *PUMA*, 5′-ACTGTGAATCC TGTGCTCTGCC-3′ and 5′- CAAATGAATGCCA GTGGTCACAC-3′; *Bax*, 5′- TCTACTTTGCCAGCAA ACTGGTGC-3′ and 5′- TGTCCAGCCCATGATGGTT CTGAT-3′; *Noxa*, 5′- CCGTGTGTAGTTGGCATCTC-3′ and 5′-CCCACTCAGCGACAGAGC-3′.

### Reporter assays

In order to determine the transcriptional activity of the promoters of *p21*, *Bax*, and *Noxa*, LNCaP cells were transiently co-transfected with pSV40 and one of three reporter constructs (p21-Luc, Bax-Luc, or Noxa-Luc) The Renilla luciferase reporter plasmid was included as an internal control. Cells were harvested, total cell extracts were prepared, and dual luciferase activity was measured according to the manufacturer's instructions (Promega). All reporter activities were normalized to *Renilla* luciferase activity and are presented as the mean (± standard deviation) of three independent experiments.

### p53 protein crosslinking assay

The stability of endogenous p53 was measured in LNCaP cells undergoing delphinidin-induced apoptosis. Cells were prepared with lysis buffer (50 mM Tris-Cl (pH 7.5), 150 mM sodium chloride, 1% NP40, 10 mM sodium fluoride, 10 mM sodium pyrophosphate, and protease inhibitors just before use) and were then incubated for 30 min on ice. For immunoprecipitation, after treatment with delphinidin for 24 h, LNCaP cells were washed and harvested with cold PBS. Cell lysates were prepared with lysis buffer (50 mM Tris-Cl (pH 7.5), 150 mM sodium chloride, 1% NP40, 10 mM sodium fluoride, 10 mM sodium pyrophosphate, and protease inhibitors) for 30 min on ice, and then centrifuged at 13,000 rpm for 10 min at 4 °C. The total cell lysate protein was incubated with anti-p53 (DO-1) (Santa Cruz Biotechnology) and 20 μL of protein A/G agarose for 2 h at 4°C. After washing three times with agarose bead washing buffer, glutaraldehyde (Sigma-Aldrich) was added to end up with three different conditions: no glutaraldehyde; glutaraldehyde at a final concentrations of 0.002%; and glutaraldehyde at a final concentration of 0.004%. After incubating for 20 min at 37 °C, the crosslinking reaction was terminated by adding 2X loading buffer. The samples were then heated at 100 °C for 5 min, separated by SDS-PAGE on 8% gels, and transferred to nitrocellulose membranes [30, 42]. The blocked membranes were incubated with anti-p53 antibody at 4 °C for 2 h. The membranes were then subjected to western blot analysis and visualized in the developer apparatus.

### Statistical analysis

Statistical analysis was performed using Student's *t*-test and the SPSS software (SPSS Inc., Chicago, IL, USA). A statistical threshold of *P* < 0.05 was considered statistically significant.
